# Attenuation of STAT3 Signaling Cascade by Daidzin Can Enhance the Apoptotic Potential of Bortezomib against Multiple Myeloma

**DOI:** 10.3390/biom10010023

**Published:** 2019-12-23

**Authors:** Min Hee Yang, Sang Hoon Jung, Arunachalam Chinnathambi, Tahani Awad Alahmadi, Sulaiman Ali Alharbi, Gautam Sethi, Kwang Seok Ahn

**Affiliations:** 1 KHU-KIST Department of Converging Science and Technology, Kyung Hee University, Seoul 02447, Korea; didmini@naver.com (M.H.Y.); shjung@kist.re.kr (S.H.J.); 2 Department of Science in Korean Medicine, Kyung Hee University, 24 Kyungheedae-ro, Dongdaemun-gu, Seoul 02447, Korea; 3 Department of Botany and Microbiology, College of Science, King Saud University, Riyadh 11451, Saudi Arabia; carunachalam@ksu.edu.sa (A.C.); sharbi@ksu.edu.sa (S.A.A.); 4 Department of Pediatrics, College of Medicine and King Khalid University Hospital, King Saud University Medical City, Riyadh 11461, Saudi Arabia; talahmadi@ksu.edu.sa; 5 Department of Pharmacology, Yong Loo Lin School of Medicine, National University of Singapore, Singapore 117600, Singapore

**Keywords:** daidzin, bortezomib, STAT3, multiple myeloma

## Abstract

Daidzin (DDZ) extracted from *Pueraria lobate* (Fabaceae) is a widely known phytoestrogen. DDZ can display anti-cancer activities against breast and prostate cancers, but its anti-oncogenic actions in multiple myeloma (MM) cells have not been studied. The signal transducer and activator of transcription 3 (STAT3) can control key processes including proliferation, differentiation, and survival in MM cells. Here, we noted that DDZ abrogated STAT3 activation (both constitutive as well as inducible) at Tyr705 and Ser727 in MM cells. Additionally, DDZ mitigated the phosphorylation of STAT3 upstream Janus-activated kinases (JAK1/2) and c-Src kinases. Pervanadate (tyrosine phosphatase blocker) exposure altered the DDZ-induced inhibition of STAT3 activation, thus affecting the action of this phytoestrogen on apoptosis. Moreover, DDZ impeded proliferation and augmented the apoptotic effects of bortezomib (Bor) in MM cells. Overall, the data indicate that DDZ may act as a potent suppressor of STAT3 signaling cascade, and the co-treatment of DDZ and Bor could be a promising therapeutic strategy, specifically in MM.

## 1. Introduction

Multiple myeloma (MM) is a lethal disease [1,2] that is marked by an abnormal proliferation of plasma cells in the bone marrow and associated features such as serum monoclonal gammopathy and immune suppression [3,4,5,6,7]. Immunomodulating agents (thalidomide) and proteasome inhibitors (bortezomib), along with other anti-inflammatory drugs, are routinely employed for MM therapy [8,9]. However, these pharmacological agents exhibit substantial toxicity, and poor patient response rates have been noted upon exposure to these drugs [10,11,12,13,14,15]. As a result, novel treatment regimens are required to circumvent the various drawbacks of existing anti-MM therapies.

Accumulating studies have indicated that the signal transducer and activator of transcription 3 (STAT3) protein can be employed as a putative target for cancer therapy [16,17,18,19,20,21]. STAT can act as an oncogene that can be overexpressed in its phosphorylated form in various human cancer cells including MM, leukemia, lymphomas, and solid tumors [1,5,22,23]. STAT3 can regulate cellular apoptosis, growth, and metastasis by affecting the transcription of various oncogenic genes [17,24,25,26,27]. The activation of STAT3 can be stimulated by various cytokines and can induce its homodimerization, nuclear localization, DNA binding, and robust transcription [1,22,23,28,29,30,31]. The phosphorylation of STAT3 can occur both at tyrosine and serine residues located at the 705 and 727 transactivation domains [24,32]. Interestingly, protein tyrosine phosphatases (PTPs) can play a critical role in regulating the STAT3 pathway. Protein tyrosine phosphatase (PTPε) can exist in two major forms: transmembrane (PTPε M) and cytosolic (PTPε C). Interestingly, PTPε C overexpression can abrogate tumor incidence within the spleen, thus suggesting that PTPε C can function as a bona fide tumor suppressor protein [33]. Moreover, SHP-1 has also been found to negatively regulate STAT3 activation [34]. Overall, the development of new pharmacological drugs that can regulate STAT3 activation and can be employed for MM therapy remains an area of significant interest.

Daidzin (DDZ) is a common phytoestrogen that can be isolated from *Pueraria lobate* (Fabaceae), which are found in soy and soy products [35,36]. DDZ has shown potential efficacy and safety as a food supplement to prevent osteoporosis [37]. DDZ has been found to bind to the estrogen receptor and to enhance the acetylcholinesterase activity of the rat neuronal cell line at low micro molar concentrations [35]. DDZ has also been implicated in the prevention of breast and prostate cancers. It can exhibit anti-proliferative activity against MCF-7 breast cancer cells by affecting their invasive potential [38,39]. In prostate cancer, DDZ may exert its anti-cancer effects at relatively early stages of prostate cancer development [40]. However, the anti-oncogenic effects of DDZ in MM have not been deciphered. As STAT3 can regulate the initiation and progression of MM, it may be possible that DDZ can alter this cascade to exert its anti-MM effects.

Here, we determined whether DDZ can affect apoptosis through the induction of PTPε and SHP-1 proteins involved in the regulation of the STAT3 pathway. We note for the first time that DDZ can significantly affect STAT3 activation by upregulating PTPε and SHP-1 tyrosine phosphatases in diverse MM cells. This inhibition negatively affects various hall marks of cancer involved in MM growth and advancement.

## 2. Materials and Methods

### 2.1. Reagents

Daidzin (DDZ) was purchased from Weikeqi Biological Technology (Chengdu, Sichuan. China). Bovine serum albumin (BSA) and 3-(4,5-dimethylthiazol-2-yl)-2,5-diphenyltetrazolium bromide (MTT) were purchased from Sigma-Aldrich (St. Louis, MO). Alexa Fluor^®^ 488 donkey anti-mouse IgG (H+L) antibody and Fluor^®^ 594 donkey anti-rabbit IgG (H+L) antibody were obtained from Life Technologies (Grand Island, NY, USA). A LightShift^®^ Chemiluminescent electrophoretic mobility shift assay (EMSA) kit was purchased from Thermo Fisher Scientific Inc. An FITC Annexin V Apoptosis Detection Kit was purchased from BD Pharmingen™ (BD Biosciences, Becton Dickinson, Franklin Lakes, NJ, USA).

### 2.2. Cell Lines and Culture Conditions

Human multiple myeloma U266 and MM1.S cells, human lung carcinoma A549 cells, human prostate carcinoma DU145 cells, human pancreatic carcinoma Panc-1 cells, human breast carcinoma MCF-7 cells, and human gastric adenocarcinoma SNU-1 cells were obtained from the American Type Culture Collection (Manassas, VA, USA). U266, MM1.S, DU145, Panc-1, MCF-7, and SNU-1 cells were cultured in an RPMI 1640 medium. A549 cells were cultured in a DMEM low glucose medium. The medium contained 10% fetal bovine serum (FBS) and 1% penicillin–streptomycin. In addition, cells were maintained at 37 ℃ under a 5% CO_2_ atmosphere.

### 2.3. Isolation of Human Peripheral Blood Mononuclear Cells (PBMCs)

Human peripheral blood mononuclear cells (PBMCs) were isolated from the blood of healthy adult donors (volunteers) via density gradient centrifugation on a Lymphoprep (Axis-Shield PoCAS, Oslo, Norway).

### 2.4. MTT Assay

Cell viability was measured with an MTT assay to detect NADH-dependent dehydrogenase activity, as outlined previously [25].

### 2.5. Western Blot Analysis

For the detection of various antibodies, cells were treated with DDZ for the indicated concentrations and time points. Then, cells were harvested and lysed with a lysis buffer (20 mM Tris (pH 7.4), 250 mM NaCl, 2 mM EDTA (pH 8.0), 0.1% Triton X-100, 0.01 mg/mL aprotinin, 0.005 mg/mL leupeptin, 0.4 mM phenyl methane sulfonyl fluoride (PMSF), and 4 mM NaVO4), and the total protein concentrations were determined by a Bradford reagent (Bio-Rad, Hercules, CA, USA). Equal amount of lysates resolved in a 10%–15% SDS-polyacrylamide gel. After SDS-PAGE, the protein was transferred onto a nitrocellulose membrane, blocked with 5% skim milk in 1× TBST (1× TBST with 0.1% Tween 20) and proved with specific primary antibodies: anti-p-STAT3(Tyr705), anti-p-Janus-activated kinase 1 (JAK1) (Tyr1022/1023), anti-JAK1, anti-p-JAK2(Tyr1007/1008), anti-JAK2, anti-p-Src(Tyr416), anti-Src, anti-cleaved caspase3, and anti-Cyclin D1, anti-STAT3, anti-PTPε, anti-SHP-1, anti-procaspase-3, anti-PARP, anti-Bcl-2, anti-Bcl-xL, anti-survivin, anti-cyclin D1, anti-COX-2, and anti-β-actin antibodies. Antibodies were incubated at 4 °C overnight. Finally, the membranes were incubated with horseradish peroxidase (HRP)-conjugated anti-rabbit IgG antibodies and anti-mouse IgG antibodies (diluted 1/5000 in TBST) at room temperature for 2 h. The membranes were detected via chemiluminescence (ECL) (EZ-Western Lumi Femto, DOGEN). After detection, the same blots were stripped for 1 h and reprobed with anti-β-actin antibodies to demonstrate equal protein loading [41].

### 2.6. Electrophoretic Mobility Shift Assay (EMSA) for STAT3-DNA Binding

STAT3-DNA binding was analyzed by an electrophoretic mobility shift assay (EMSA), which was performed as elaborated in detail before by our group [42].

### 2.7. Immunocytochemistry

After U266 cells were treated with 30 μM of DDZ for 3 h, the cells were fixed with 4% paraformaldehyde (PFA) at room temperature for 20 min and washed three times by 1× PBS. The cells were permeabilized with 0.2% Triton-X 100 for 10 min and blocked with 5% BSA in PBS for 1 h. After that, the cells were incubated with anti-p-STAT3(Tyr705), anti-p-STAT3(727), and anti-STAT3 (1:100) for overnight at 4 °C. The next day, cell were washed three times by 1× PBS and incubated with Alexa Fluor^®^ 488 donkey anti-mouse IgG (H+L) antibody and with Alexa Fluor^®^ 594 donkey anti-rabbit IgG (H+L) secondary antibodies (1:1000) at room temperature for 1 h. Then, they were stained with DAPI (1 μg/mL) for 3 min at room temperature and mounted in a Fluorescent Mounting Medium (Golden Bridge International Labs, Mukilteo, WA, USA). Finally, the fluorescence signal was detected by using an Olympus FluoView FV1000 confocal microscope (Tokyo, Japan).

### 2.8. STAT3 Luciferase Reporter Assay

MM1.S cells (2 × 10^6^ cells/well) were transfected with STAT3 luciferase DNA (300 ng) and STAT3 dominant negative DNA (300 ng) by using a NEON^TM^ Transfection system (Invitrogen, Carlsbad, CA, USA), and the reporter assay was done as outlined before [42].

### 2.9. Cell Transfection with PTPε siRNA and SHP-1 siRNA 

U266 cells were transfected with 100 nM of PTPε or SHP-1 siRNAs, as described previously [43].

### 2.10. Cell Cycle Analysis

To determine the effects of DDZ on cell cycle progression, a cell cycle analysis was performed by using propidium iodide, as described earlier [44].

### 2.11. Annexin V Assay

An annexin V assay was performed to determined apoptosis by using an Annexin V Apoptosis Detection Kit (BD Biosciences, Becton Dickinson, Franklin Lakes, NJ, USA) as outlined earlier [45].

### 2.12. TUNEL Assay

U266 cells were treated with 30 μM of DDZ for 24 h, and MM1.S cells were pre-treated with 30 μM of DDZ for 3 h and treated with IL-6 (10 ng/mL) for 21 h. Late apoptotic cell death was determined with a Roche Diagnosis TUNEL assay kit, as described before [44].

### 2.13. Reverse Transcription Polymerase Chain Reaction (RT-PCR)

U266 cells were treated with DDZ (0, 10, 20, and 30 μM) for 3 h. Total RNA was extracted with Trizol reagent. RNA purified with chloroform, and isopropanol RNA was converted to cDNA by using superscript reverse transcriptase and Taq polymerase by reverse transcription polymerase chain reaction (RT-PCR) (TAKARA, Tokyo, Japan). The relative expressions of PTPε C (5’-GGAGGAGGAGTTCAGGAAATTG-3’ and 5’-CTGGGTGGCGATGAAATAGT-3’), PTPε M (5’-CTTGCAGCCTACTTCTTCA-3’ and 5’-TTGAACTCCTCTCGGAACCG-3’) and SHP-1 (5’-GGCTTCTGGGAGGAGTTTGAG-3’ and 5’-CGGAGTTTGTATTCGGTTGTG-3’) were analyzed by using PCR. Glyceraldehyde-3-phosphate dehydrogenase (GAPDH) was used as an loading control. Then PCR products were run on 1% agarose gel and stained with Loading Star (Dynebio, Seongnam, Korea). Stained bands were detected with UV light [46].

### 2.14. Combination Therapy with DDZ and Bortezomib

To analyze the combination effect of DDZ and bortezomib (Bor), U266 cells were seeded and co-treated with DDZ and Bor with various concentrations for 24 h. Cytotoxicity was analyzed by an MTT assay to find optimal rate of drugs, and then the cells were evaluated by the CalcuSyn (BIOSOFT, Ferguson, MO, United States) software. We input each data point to calculate a combination index (CI) and to select a moderate combination rate. This was then used to evaluate synergy and antagonism: CI < 1, CI = 1, and CI >1, respectively [18,43,47].

### 2.15. Live and Dead Assay

To determine cytotoxicity, we performed Live and Dead assays, as described earlier [48].

### 2.16. Statistical Analysis

The results are expressed as means ± SD, and an analysis of variance (ANOVA) with Bonferroni’s test was used for the statistical analysis of multiple comparisons of data. A *p*-value of 0.05 or less was considered as significant.

## 3. Results

### 3.1. DDZ Suppresses the Cell Viability in MM Cells

**Figure 1:**
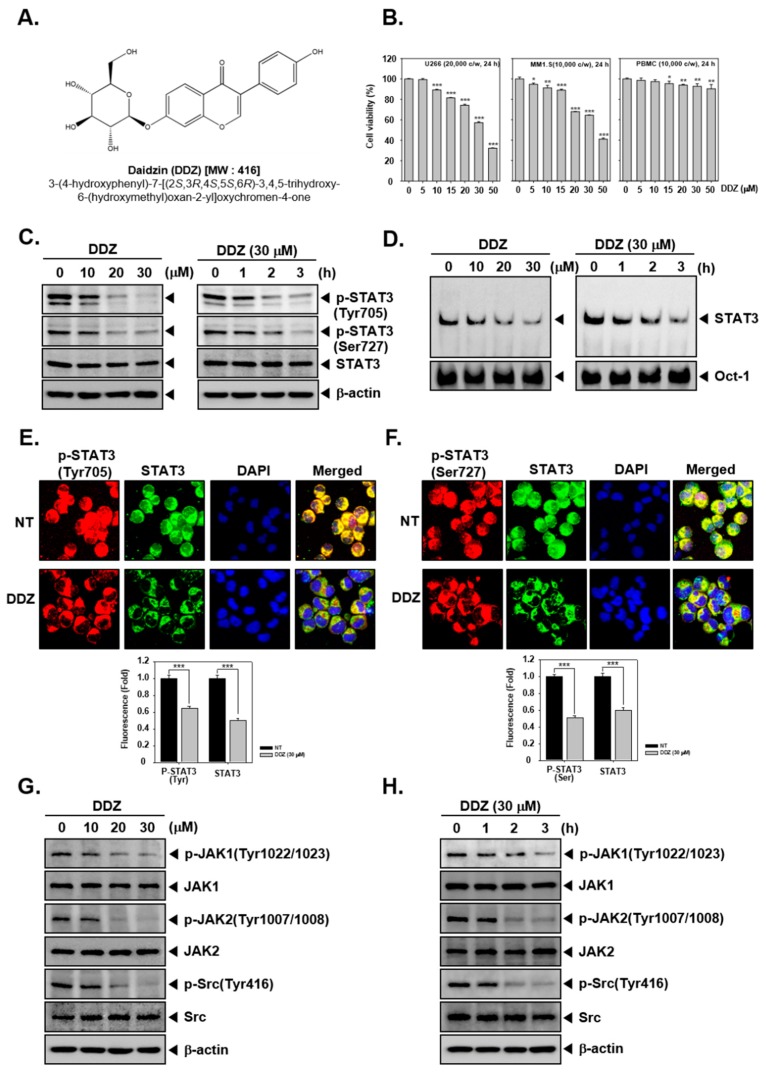
Daidzin (DDZ) suppresses constitutive STAT3 (signal transducer and activator of transcription 3) activation in U266 cells. (**A**) The chemical structure of DDZ. (**B**) To determine the cell viability, a 3-(4,5-dimethylthiazol-2-yl)-2,5-diphenyltetrazolium bromide (MTT) assay was employed. U266 cells (2 × 10^4^ cells/well) were treated with indicated concentrations for 24 h. (**C**) U266 cells were treated with dose dependent (0, 10, 20, and 30 µM) or time dependent (0, 1, 2, and 3 h) conditions, and western blotting was done. (**D**) U266 cells were treated as described above in panel (**C**), and nuclear STAT3 levels were measured by an electrophoretic mobility shift assay (EMSA). (**E**) and (**F**) U266 cells (1 × 10^6^ cells/well) were treated with 30 µM of DDZ for 3 h, and intracellular p-STAT3(Tyr705) and p-STAT3(Ser727) distribution was analyzed by immunocytochemistry. (**G**)–(**H**) U266 cells (1 × 10^6^ cells/well) were treated as described above in panel (**C**), and a western blot analysis was performed. The results shown are representative of three independent experiments. The results are presented as the mean ± SD. * *p* < 0.05, ** *p* < 0.01, *** *p* < 0.001 compared the control.

The structure of daidzin (DDZ) is illustrated in Figure 1A. The cytotoxic action of DDZ towards MM cells was examined via an MTT assay. It was noted that this phytoestrogen attenuated the viability of U266 and MM1.S cells in a dose-dependent fashion, but PBMCs maintained their viability (Figure 1B).

### 3.2. DDZ Affects STAT3 Activation in U266 Cells

We next determined if DDZ could alter STAT3 activation in U266 cells. As depicted in Figure 1C, DDZ substantially suppressed the constitutive activation of both p-STAT3 (Tyr705) and p-STAT3(Ser727) in U266 cells. We next examined whether DDZ could affect the ability of STAT3 to bind to DNA, and it was noted that DDZ could effectively modify the DNA-binding activity of STAT3 (Figure 1D). Additionally, we examined the effect of DDZ to regulate the nuclear localization of STAT3 in U266 cells by immunocytochemistry. Figure 1E,F demonstrate that DDZ could reduce the translocation of phospho-STAT3 and total STAT3 to the nucleus.

### 3.3. DDZ Modulates Activation of Upstream Kinases

To identify the mechanism by which DDZ affected STAT3 signaling, we examined the levels of kinases involved in regulating this pathway. As shown in Figure 1G,H, the basal levels of the JAK1/2 and Src kinases were substantially reduced upon DDZ exposure.

### 3.4. DDZ Alters Inducible STAT3 Activation

**Figure 2:**
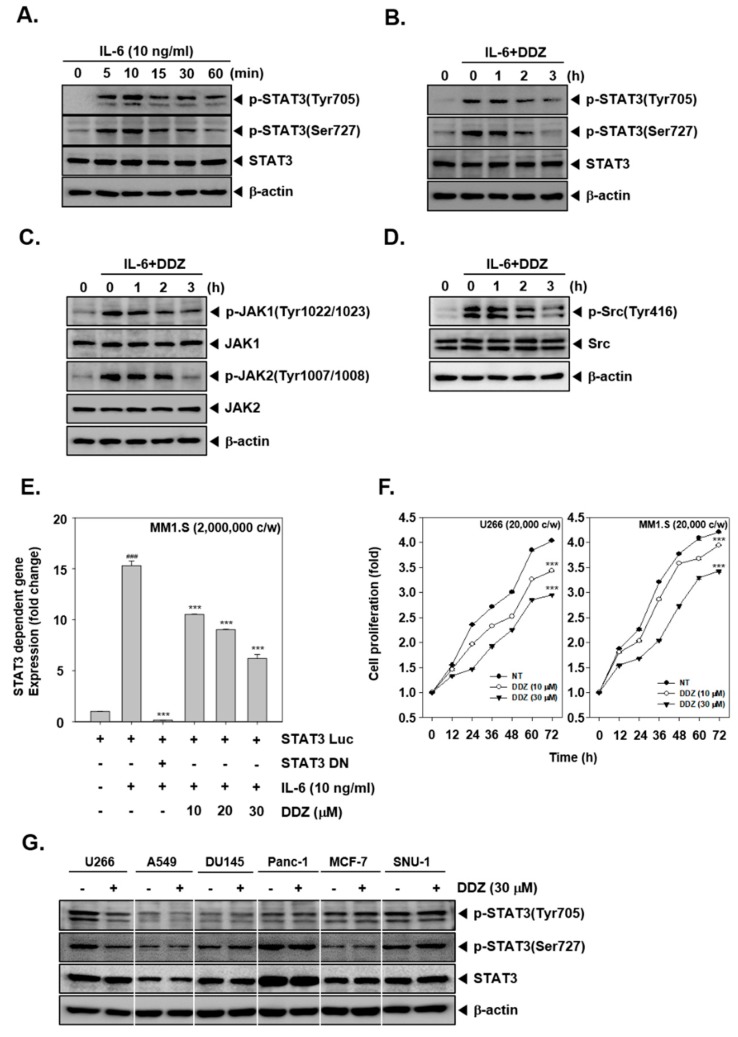
DDZ inhibited IL-6-induced STAT3 activation in human multiple myeloma MM1.S cells. (**A**) MM1.S cells (1 × 10^6^ cells/well) were stimulated with IL-6 (10 ng/mL) for the indicated time. Whole cell lysates were prepared, and western blotting was carried out against various proteins. (**B**)–(**D**) MM1.S cells (1 × 10^6^ cells/well) were treated with 30 μM of DDZ for 0, 1, 2, and 3 h and then stimulated with IL-6 (10 ng/mL) for 10 min. Thereafter, western blotting was done. (**E**) MM1.S cells (2 × 10^6^ cells/well) were transfected with STAT3-luciferase plasmid for 48 h, treated with DDZ (0–30 µM) for 3 h, and then stimulated with IL-6 (10 ng/mL) for 10 min. Thereafter, luciferase activity was measured. (**F**) A cell proliferation study was performed with an MTT assay. U266 and MM1.S cells (2 × 10^4^ cells/well) were seeded onto a 96-well plate and incubated with 30 µM of DDZ for indicated time intervals. The results are presented as the mean ± SD. *** *p* < 0.001 compared the control. (**G**) U266, A549, DU145, Panc-1, MCF-7, and SNU-1 cells were treated with 30 μM of DDZ for 3 h, and western blotting was done. The results shown are representative of three independent experiments.

We found that IL-6 activated the phosphorylation of STAT3 (Tyr705 and Ser727); this response was maximum at 10 min, and then it decreased (Figure 2A). Next, MM1.S cells were treated with 30 µM of DDZ for 0, 1, 2, and 3 h and stimulated with IL-6 (10 ng/mL) for 10 min, and cell lysates were subjected to western blotting. The results indicated that DDZ altered IL-6-induced STAT3 (Tyr705 and Ser727) phosphorylation as well as JAK1/2 and Src phosphorylation in MM1.S cells. To analyze the effect of DDZ on gene transcription, cells were transfected with a STAT3-luciferase construct and a STAT3 dominant-negative construct; thereafter. luciferase activity was monitored. As shown in Figure 2E, in DDZ pretreated cells, IL-6-induced STAT3 activity was significantly attenuated.

### 3.5. DDZ Suppressed the Proliferation of MM Cells

U266 and MM1.S cells were exposed to varying concentrations of DDZ for 72 h, and their proliferation rate was analyzed. As shown in Figure 2F, DDZ attenuated the proliferation ability in U266 and MM1.S cells as compared to non-treated cells (*** *p* < 0.001).

### 3.6. DDZ Does Not Affect STAT3 Phosphorylation in Other Tumor Cell Lines

We analyzed if DDZ could also affect STAT3 activation in other solid tumors. As shown in Figure 2G, DDZ negatively affected p-STAT3 levels in U266 cells but not in A549, DU145, Panc-1, MCF-7, and SNU-1 cells. 

### 3.7. Tyrosine Phosphatases Can Regulate STAT3 Modulatory Actions of DDZ

**Figure 3:**
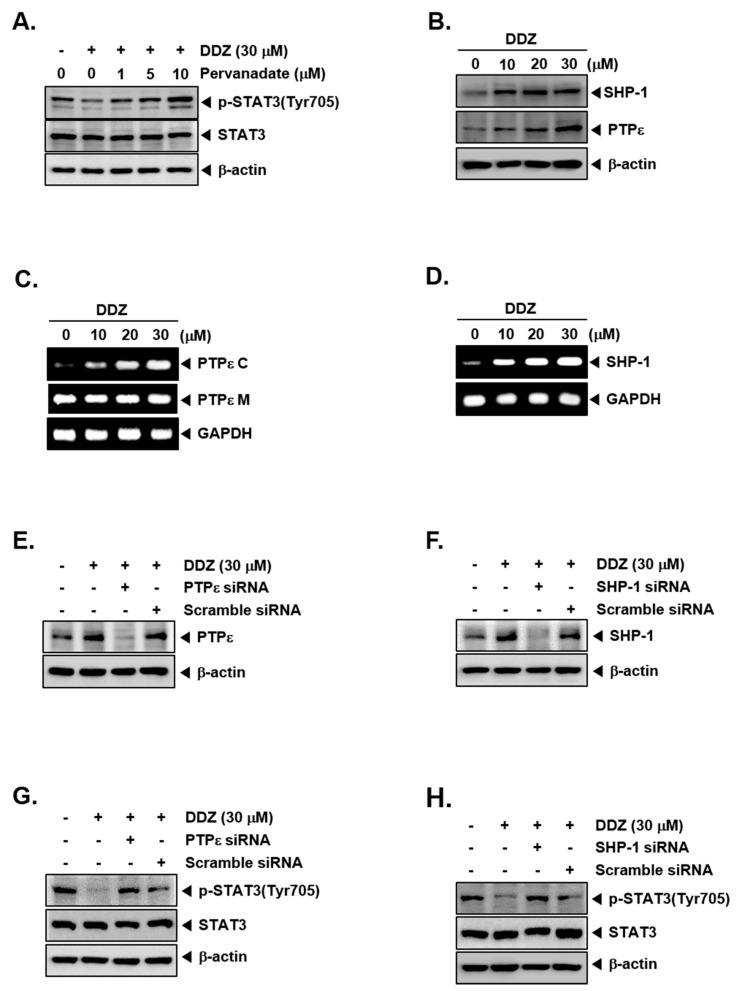
DDZ increased protein tyrosine phosphatases protein tyrosine phosphatase (PTPε) and SHP-1 levels. (**A**) U266 cells (1 × 10^6^ cells/well) were treated with various concentrations of pervanadate and 30 μM of DDZ for 3 h. The expression of various proteins was analyzed by western blotting. (**B**) U266 cells (1 × 10^6^ cells/well) were treated with the indicated concentrations of DDZ for 3 h, and western blotting was done. (**C**) and (**D**) U266 cells (1 × 10^6^ cells/well) were treated as described above in panel (**B**), and total RNA was extracted and examined for the expression of various genes. (**E**)–(**H**) U266 cells (2 × 10^6^ cells/well) were transfected with scrambled or PTPε- or SHP-1-specific siRNA (100 nM). After 24 h, cells were treated with 30 μM of DDZ for 3 h, and the western blot analysis was done. The results shown are representative of three independent experiments.

**Figure 4:**
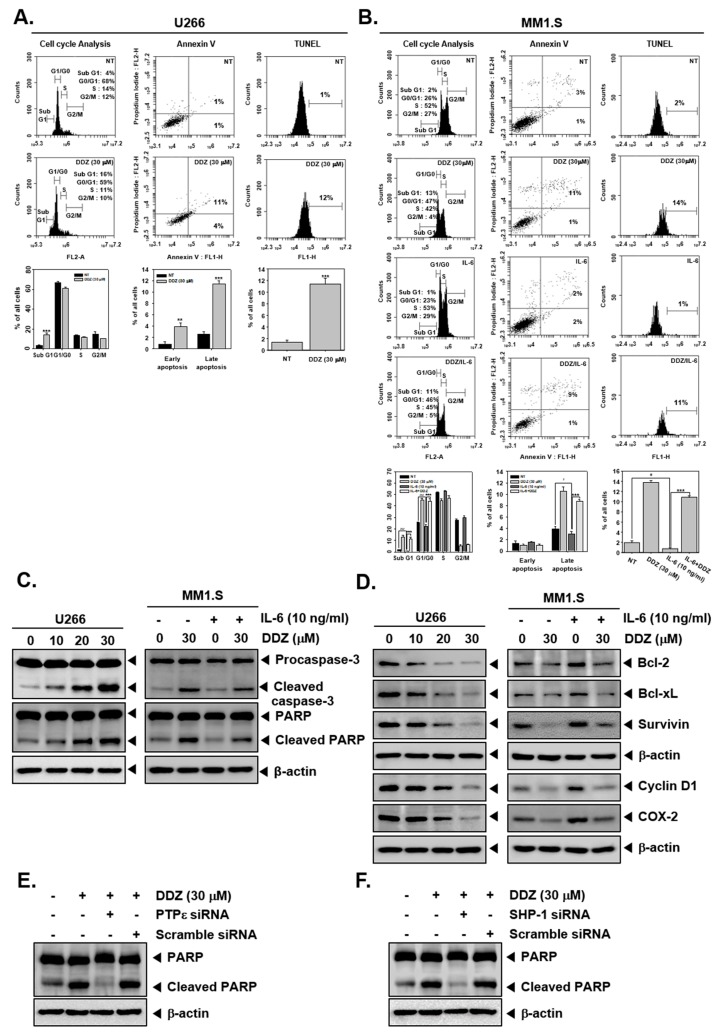
DDZ promotes apoptosis in multiple myeloma (MM) cells. (**A**, **B**) U266 cells were treated with 30 μM of DDZ for 24 h, and MM1.S cells were treated with 30 μM of DDZ for 24 h and stimulated with IL-6 (10 ng/mL) for 10 min. Cell cycle analysis: The cells were fixed with 100% ethanol, and cell cycle analysis was done using cytometry. Annexin V: cells were stained with Annexin V FITC and PI for 15 min before being analyzed by flow cytometry. TUNEL: The cells were fixed by 4% PFA (paraformaldehyde), stained with a TUNEL assay reagent, and analyzed with flow cytometry. The results are presented as the mean ± SD. ** *p* < 0.01, *** *p* < 0.001, ^#^ *p* < 0.05, ^###^ *p* < 0.001 compared the control. (**C**, **D**) U266 cells (1 × 10^6^ cells/well) were treated with indicated concentrations of DDZ for 24 h, and various proteins were examined by western blot analysis. MM1.S cells (1 × 10^6^ cells/well) were treated with 30 μM of DDZ for 24 h and stimulated with IL-6 (10 ng/mL) for 10 min. Then various proteins were examined by western blot analysis. (**E**, **F**) U266 cells (2 × 10^6^ cells/well) were transfected with scrambled or PTPε- or SHP-1-specific siRNA (100 nM). After 24 h, cells were treated with 30 μM of DDZ for 24 h. Whole cell lysates were prepared and analyzed by western blotting against the PARP protein.

We investigated if the STAT3 attenuation caused by DDZ involved the role of protein tyrosine phosphatases (PTPs). Interestingly, sodium pervanadate altered the mitigation of STAT3 activation (Figure 3A), suggesting that this action of DDZ may be mediated through tyrosine phosphatase. Protein tyrosine phosphatases (PTPs) have been reported as STAT activation regulators [34,48,49]. We investigated whether DDZ could affect the protein levels of PTPε and SHP-1 in U266 cells. As shown in Figure 3B, DDZ induced PTPε and SHP-1 expression both at the protein and mRNA levels (Figure 3C,D). We performed PTPε and SHP-1 knockdowns to confirm whether the abrogation of constitutive STAT3 phosphorylation by DDZ occurred through PTPε and SHP-1. We observed that DDZ-induced PTPε and SHP-1 expression was indeed suppressed in the cells transfected with their respective siRNAs, but it was not altered in those treated with scrambled siRNA (Figure 2E,F). As shown in Figure 2G,H, the deletion of PTPε and SHP-1 modulated the inhibition of STAT3 phosphorylation and the induction of PARP cleavage by DDZ (Figure 4E,F).

### 3.8. DDZ Promotes Apoptotic Cell Death in MM Cells

A wide variety of biochemical techniques were used to decipher the apoptotic actions of DDZ. As shown in Figure 4A,B, DDZ treatment caused an accumulation of cells (from 3% to 14%) in the SubG1 phase. Moreover, DDZ increased the content of SubG1 (2%→13%) and G0/G1 phases (26%→47%) in MM1.S cells. Late apoptosis was induced (1%→11%), and its peak was shifted to the right side (approximately 1%→12%) with an increase in apoptosis in U266 cells (Figure 4A, Annexin V and TUNEL assay section). Additionally, late apoptosis was induced to a lesser extent in IL-6-treated MM1.S cells (3%→2%) (Figure 4B, Annexin V and TUNEL assay section). Additionally, it was observed that DDZ treatment promoted caspase-3 activation and PARP cleavage (Figure 4C, left panel), whereas IL-6 treatment reduced the activation of caspase-3 and PARP cleavage (Figure 4C, right panel). Moreover, DDZ substantially modulated the levels of various proteins involved in cell survival, proliferation, and angiogenesis in MM cells (Figure 4D).

### 3.9. DDZ Enhances the Anti-Cancer Actions of Bortezomib

**Figure 5:**
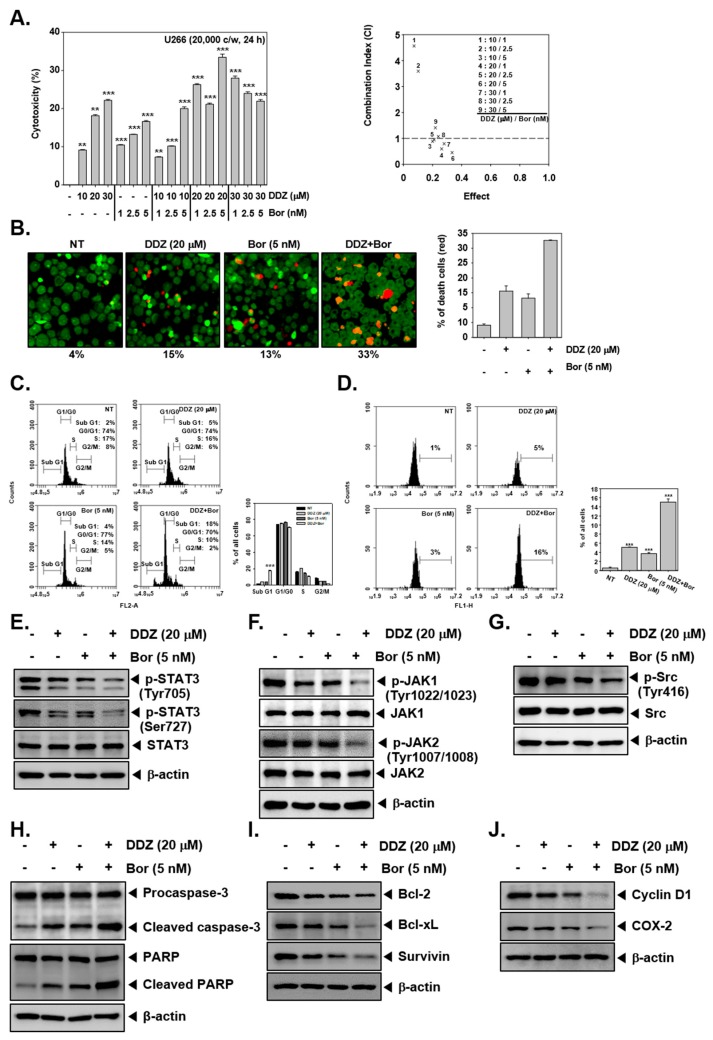
DDZ and bortezomib (Bor) could augment apoptotic effects. (**A**) To determine the effect of DDZ and bortezomib (Bor) on cytotoxicity, an MTT assay was done. U266 cells (2 × 10^6^ cells/well) were treated with DDZ and Bor at the indicated concentrations for 24 h. The average of the combination index (CI) values was calculated for mine separate combinations. A CI lower than 1 was regarded as synergistic, a CI of 1 was regarded as additive, and a CI higher than 1 was regarded as antagonistic. The average of CI values for various combinations suggests that the best combination ratio was 20 μM of DDZ and 5 nM of Bor. (**B**) Live and Dead assays were performed to determine the combinatory effects of DDZ and Bor. U266 cells were treated as described above. Live cells were stained in green, and dead cells were stained in red. The graph (right) shows the rate of dead cells by quantification. (**C**) U266 cells were treated with 20 μM of DDZ and 5 nM of Bor for 24 h, and then the cells were analyzed by flow cytometry. (**D**) The cells were treated as described above, then stained with TUNEL assay reagent, and analyzed with flow cytometry. (**E**)–(**G**) U266 cells (1 × 10^6^ cells/well) were treated with 20 μM of DDZ and 5 nM of Bor for 3 h. Whole cell lysates were prepared, and western blotting for various proteins was done. (**H**)–(**J**) U266 cells (1 × 10^6^ cells/well) were treated as described above in panel (**C**), and western blot analysis was carried out. The results are presented as the mean ± SD. *** *p* < 0.001 compared the control.

U266 cells were treated with various combinations of DDZ and Bor for 24 h. As shown in Figure 5A, 20 μM of DDZ. and 5 nM of Bor synergistically attenuated cellular growth. Next, we determined cell viability with Live and Dead assays. The DDZ and Bor co-treatment induced higher cell death as compared to the single treatment alone (Figure 5B). Next, SubG1 phase arrest was increased in the DDZ and Bor combination treatment compared with non-treated cells and in response to single treatment. Additionally, the DDZ and Bor combination treatment significantly increased apoptosis, as demonstrated by the TUNEL assay. As shown in Figure 5E–G, the DDZ and Bor combination treatment abrogated STAT3 phosphorylation and upstream signaling kinases to a greater extent than the single treatment. Moreover, the combination treatment also increased caspase-3 activation and PARP cleavage, and it caused a reduction in the levels of various oncogenic apoptotic proteins.

## 4. Discussion

Previously, DDZ has been reported to exhibit anti-neoplastic actions effects against breast and colon cancers [38,39,40]; however, no studies have discussed its activity and possible mechanism(s) in MM cells. This report aimed to analyze whether DDZ could affect oncogenesis by regulating the negative regulators of the STAT3 signaling pathway and cause the enhancement of bortezomib-induced apoptosis in MM cells. It was noted that DDZ abrogated basal and IL-6-induced STAT3 activation, abrogated the mitigation of the phosphorylation of upstream the JAK1/2 and c-Src kinases, and promoted the induction of PTPε and SHP-1 proteins. This disruption in STAT3 signaling cascade could cause a significant reduction in the expression of various oncogenic gene products, stimulate apoptosis and mitigate cellular growth. DDZ also augmented the apoptotic actions of Bor, as demonstrated by various biochemical assays.

It was observed that DDZ could inhibit STAT3 phosphorylation at both tyrosine 705 and serine 727 residues in MM cells. Interestingly, these observations were restricted to MM cells, as DDZ had a negligible effect on STAT3 phosphorylation in the A549, DU145, Panc-1, MCF-7, and SNU-1 cell lines. Additionally, we noted that DDZ abrogated the DNA-binding potential and nuclear localization of STAT3. How DDZ inhibits the activation of STAT3 was mechanistically studied. The activation of JAK can promote robust STAT3 phosphorylation in different tumor cell lines, including MM [28,49]. It was found that DDZ could indeed abrogate basal JAK1, JAK2, and c-Src activation in U266 cells, as well as the inducible activation of these kinases in MM1.S cells. In addition, the inhibitory effect of DDZ on IL-6-induced STAT3 activation may be associated with its potential beneficial application against autoimmune diseases. IL-6 inhibitors have been used in the treatment of rheumatoid arthritis [50], and DDZ’s metabolite equol has been reported to mitigate microglial activation and potentiate neuroprotection in central nervous system cells [51]. Current drugs used for multiple sclerosis and other neurodegenerative disorders have efficacy but may display numerous side effects [52]. Hence, DDZ and its metabolites can serve as a novel therapeutic options against neurodegenerative and neuroinflammatory diseases. 

Various PTPs have been reported to interfere STAT3 phosphorylation [32,53]. Here, we demonstrated that that the DDZ-induced inhibition of STAT3 activation may be caused by the modulation of PTPs, namely PTPε and SHP-1. PTPε and SHP-1 can function as negative regulators of STAT3 activation [1,23,54,55]. Interestingly, DDZ could substantially promote the expression of PTPε and SHP-1 proteins and mRNA levels, but the silencing of these phosphatases could alter the DDZ-mediated abrogation of STAT3 activation and cellular apoptosis. It has been suggested that various pharmacological agents may exert their effects through the up-regulation of PTPε [23,43] as well as SHP-1 [1,6,56] expression. Interestingly, DDZ was found to simultaneously promote PTPε and SHP-1 expression, thereby clearly indicating that the PTPε and SHP-1 play an important role in the down-regulation of STAT3 by DDZ.

It was also found that DDZ could mitigate the levels of various proteins that regulate various cancer hall marks in MM cells. Aberrant STAT3 phosphorylation can regulate the process of programmed cell death by regulating the expression of pro-survival proteins [57,58]. The decrease in the levels of Bcl-2, Bcl-xL, and survivin could have promoted the apoptotic actions of DDZ. Alternatively, answering the question of whether other mechanism(s) are involved in pro-apoptotic effects requires additional experiments. Bor is routinely used for the treatment of MM cells [59], but it can exhibit severe side effects such as peripheral neuropathy, and patients frequently develop chemoresistance [10,60,61]. We further demonstrated that DDZ enhanced the apoptotic and cytotoxic actions of Bor by affecting STAT3 signaling cascade in MM cells. The combination treatment of DDZ (20 μM) and Bor (5 nM) could substantially reduce the activation of STAT3 and its upstream kinases (JAK1, JAK2, and c-Src). Additionally, the co-treatment promoted apoptosis through the suppression of diverse oncogenic gene products. Thus, the co-treatment of DDZ and Bor appears to have greater therapeutic effects than an individual treatment and can be used to mitigate the side effects of targeted therapies in MM treatment. 

Overall, our findings indicate that DDZ can effectively modulate STAT3 signaling cascade by affecting the levels of PTPε and SHP-1 proteins in MM cells. It can also significantly enhance the apoptotic effects of targeted therapies used for MM treatment. Though further studies are required in preclinical settings, our results support the possibility that the co-treatment of DDZ and Bor may exhibit greater anti-cancer effects in MM patients.
